# By Chance, Not Design: A New Furosemide Derivative From Zinc(II) Complex Studies

**DOI:** 10.1021/acsomega.5c12211

**Published:** 2026-02-05

**Authors:** Nina Podjed Rihtaršič, Romana Cerc Korošec, Barbara Modec

**Affiliations:** Faculty of Chemistry and Chemical Technology, 37663 University of Ljubljana, Večna pot 113, Ljubljana 1000, Slovenia

## Abstract

Furosemide, a sulfonamide-based diuretic, may also be of interest as a ligand capable of binding to metal ions. Our studies of zinc­(II) coordination chemistry with furosemide in organic solvents resulted in a crystalline precipitate. Initial characterization was challenging because single crystals were of insufficient quality for an unambiguous structural determination. X-ray analysis confirmed a four-coordinate zinc­(II) complex with two deprotonated furosemides (fur^–^) bound in a monodentate manner and two simple monodentate ligands. Complementary techniques, i.e., infrared spectroscopy, NMR spectroscopy, and thermal analysis, revealed their identity as ammonia, generated *in situ* from acetonitrile hydrolysis. The composition of the product is thus [Zn­(fur)_2_(NH_3_)_2_]·(CH_3_CH_2_)_2_O (**1**). Although our attempt to prepare better-diffracting crystals was unsuccessful, it led to the serendipitous discovery of a new furosemide derivative, labeled **2**. The derivative features two structural modifications compared to the parent furosemide, namely, ester and amidine functionalities. Single-crystal analysis of **2** showed the esterification of the carboxyl group with methanol and nucleophilic addition of the sulfonamide NH_2_ group to the triple bond of acetonitrile, used as a solvent. These results expand the structural chemistry of furosemide and demonstrate its capacity to undergo unprecedented transformations in zinc­(II)-mediated systems.

## Graphical abstract

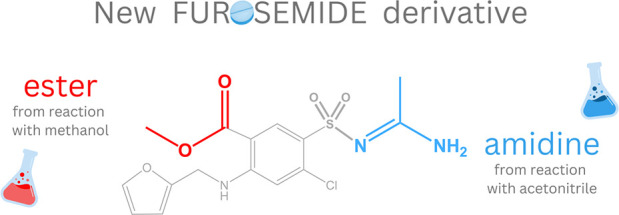

## Introduction

Furosemide, 4-chloro-2-(furan-2-ylmethylamino)-5-sulfamoylbenzoic acid (Scheme 1), is a well-known loop diuretic and a member of the sulfonamide class of compounds. It has been used for decades to reduce extracellular fluid volume in patients with heart or kidney diseases and to treat hypertension.^1,2^ Although highly effective, furosemide exhibits very low solubility in water (<0.1 mg/mL), which increases with the pH value of the medium.^1^ Other strategies to improve the solubility and bioavailability include salt formation and cocrystallization.^3,4^ Our previous research dealt with the relatively unexplored field of coordination chemistry of the furosemide anion and transition metal ions. At the time, only two coordination compounds with deprotonated furosemide (fur^–^) were reported in the Cambridge Structural Database, CSD.^5^ First, we focused on the preparation of zinc­(II) compounds with the furosemide anion.^6^ Zinc­(II) was chosen because of its essentiality for humans and catalytic properties,^7–9^ while furosemide was selected for its sulfonamide fragment,^10^ a structural motif reported to exhibit antibacterial activity.^11^ [Zn­(NH_3_)_2_(fur)_2_] was prepared by reacting zinc­(II) oxide or zinc­(II) chloride with furosemide in an aqueous ammonia solution.^6^ Our choice of metal and ligand proved to be justified, as the resulting compounds showed moderate activity against *S. epidermidis*,^6^ thus offering a promising starting point for further studies.

**Scheme 1:**
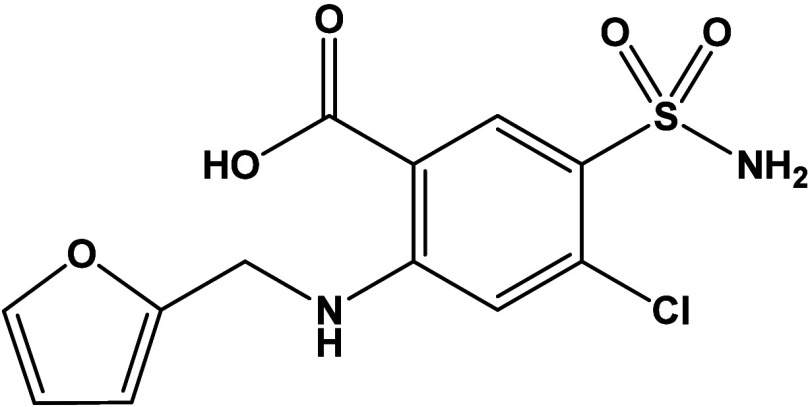
Structural Formula of Furosemide (furH)

## Results and Discussion

Interestingly, the solvothermal reaction of zinc­(II) oxide and furosemide in organic solvents yielded only a microcrystalline solid. Our aim was to elucidate its composition and structure, which we propose as [Zn­(fur)_2_(NH_3_)_2_]·(CH_3_CH_2_)_2_O (**1**). Several lines of evidence support this composition. The ^1^H NMR spectrum displays seven resonances corresponding to the protons of deprotonated furosemide, along with signals attributed to diethyl ether molecules (quartet at 3.38 ppm and triplet at 1.09 ppm). As expected, the resonance associated with exchangeable NH_2_ protons is absent. The integral ratio of diethyl ether to complex is less than one due to partial loss of solvent molecules upon removal of the product from the mother liquor. Notably, the furosemide part of the spectrum of **1** is identical to that of the previously reported complex [Zn­(fur)_2_(NH_3_)_2_].^6^ However, the similarity alone does not conclusively confirm identical composition since both spectra lack resonances for ammonia protons. This leaves open the possibility that the auxiliary ligand is water rather than ammonia. This ambiguity was resolved by thermal analysis. The sample was dried before these measurements to remove the diethyl ether molecules. Both TG-FTIR and TG-MS analyses confirmed the presence of ammonia. The TG-MS curves (Figure 1) show that during the first step (from room temperature to about 90 °C) physiosorbed water and ammonia are released from the sample, while during the second and third mass loss (temperature range 90–220 °C) ammonia is the main gaseous product. According to the NIST database, the most pronounced signal for ammonia is 17 *m*/*z* (NH_3_^+^), followed by 16 *m*/*z* (NH_2_^+^).^12^ However, strong ionization in the quadrupole mass spectrometer can alter the relative intensities of these signals. The shape of 16 and 17 *m*/*z* coincides, 15 *m*/*z* is also observed, while 18 *m*/*z* is negligible. The release of ammonia is also confirmed by TG-FTIR analysis (ESI, Figure S6). Since previous detailed thermal studies on furosemide have shown that ammonia is not released during its decomposition,^13^ we conclude that the coordinated ligand in **1** is indeed ammonia.

**Figure 1:**
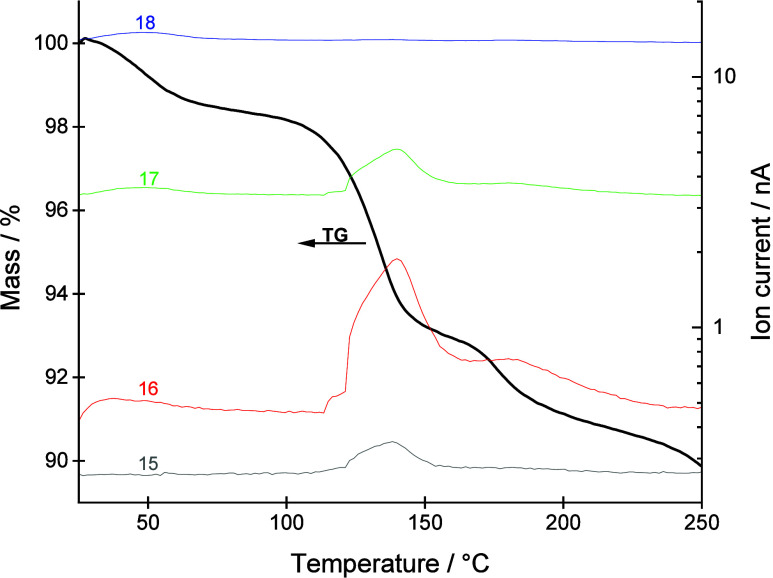
TG-MS curves under a nitrogen flow.

The infrared spectrum of compound **1** closely matches that of the known complex [Zn­(fur)_2_(NH_3_)_2_], thus providing additional confirmation of its composition.^6^ The most intense absorption bands have their origin in different vibrations within the furosemide anion: 1609 [ν_as_(COO^–^)], 1382 [ν_s_(COO^–^)], 1325 [ν_as_(SO_2_)], 1157 [ν_s_(SO_2_)], and 589 cm^–1^ (not assigned).^6,14^ The ν­(N–H) spectral region reveals a series of weak bands [3407, 3365, 3345, and 3284 cm^–1^]. Some of these correspond to coordinated ammonia, whose presence is further corroborated by a medium-intensity band at 1250 cm^–1^. A group of weak ν­(C–H) bands at 2976–2881 cm^–1^ can be ascribed to diethyl ether solvent molecules. The vibrations of the −CH_2_– structural element, present in the furosemide anion, appear in the same spectral region, yet the spectrum of the known [Zn­(fur)_2_(NH_3_)_2_] lacks bands in this region.^6^

Crystal growth of **1** was challenging and required an extended period of time. Unfortunately, the crystals obtained allowed only diffraction data of limited quality. Nevertheless, XRD revealed a tetrahedral zinc­(II) complex with two furosemide anions. The ORTEP drawing of the [Zn(fur)_2_(NH_3_)_2_] complex in **1** is shown in Figure 2, while the relevant geometric parameters are summarized in Table 1. The zinc­(II) ion is surrounded by two deprotonated furosemide ligands, each bound in a monodentate manner *via* carboxylate oxygen and by two ammonia molecules. The geometry of this four-coordinate complex is almost tetrahedral (τ_4_ = 0.88).^15^ **1** can be compared with the previously reported [Zn(fur)_2_(NH_3_)_2_] complex.^6^ Due to the presence of diethyl ether molecules in **1**, these two compounds cannot be regarded as polymorphs. There are, however, some differences in the solid-state structures of the two complex molecules (ESI, Figure S1). In the previously known compound, only half of a molecule is present in the asymmetric unit, with the other half generated by a 2-fold rotation axis. As a result, the furosemide anions have their furan rings pointing in opposite directions. In contrast, in **1**, the entire molecule is present in the asymmetric unit, with both furosemide anions having their furan rings oriented in the same direction. With the two [Zn(fur)_2_(NH_3_)_2_] molecules differing in the orientation of their furosemide ligands, they may be classified as conformational isomers.^16^ The complex molecules are linked through an intricate network of hydrogen bonds, forming supramolecular layers that stack along the *a*-axis (ESI, Figures S2 and S3). Pockets within these layers accommodate diethyl ether molecules, which are weakly hydrogen bonded (NH_3_···O = 3.053(15) Å). The solid-state structure of the previously known [Zn­(fur)_2_(NH_3_)_2_] also displays supramolecular layers.^6^ However, a different orientation of the deprotonated furosemide ligands in **1,** compared to the known complex, results in a different packing arrangement of complex molecules in the solid state.

**Figure 2:**
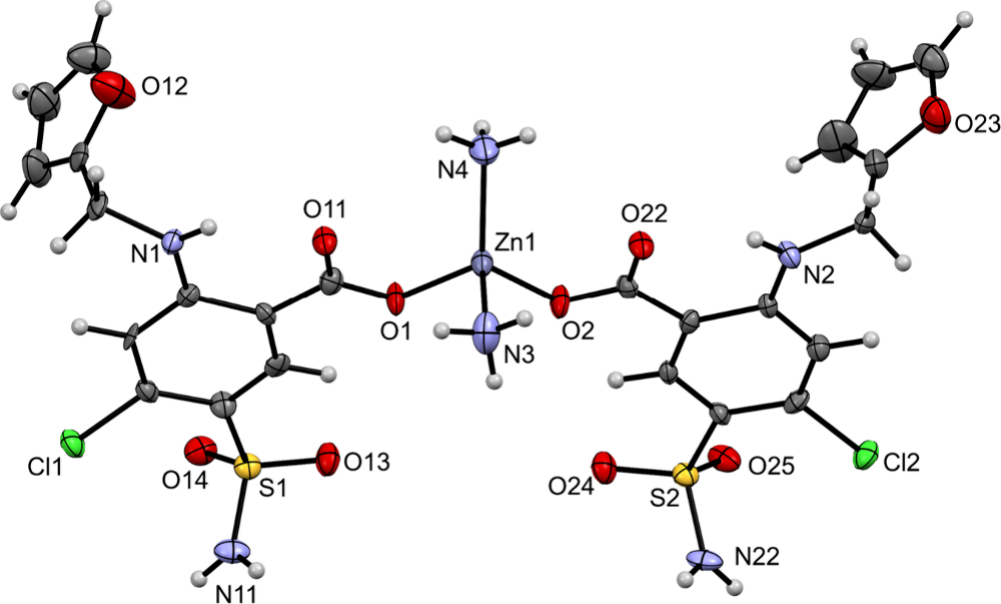
ORTEP drawing of [Zn­(fur)_2_(NH_3_)_2_] in **1**. The displacement ellipsoids are shown at the 50% probability level. Hydrogen atoms are shown as spheres of arbitrary radii.

**Table 1:** Relevant Geometric Parameters [Å, °] for Complex in **1**

Zn–O	Zn–N	τ_4_
1.954(5), 1.958(5)	2.007(8), 2.024(9)	0.88

After the identification of **1**, the source of ammonia needed to be determined. Acetonitrile is known to undergo hydrolysis at elevated temperatures,^9,17–19^ producing, among other compounds, ammonia. However, under our reaction conditions, this conversion is neither quantitative nor easily controlled. The initial step of hydrolysis yields acetamide, which subsequently hydrolyzes to produce acetate or acetic acid (depending on the pH) and ammonia. Formation of acetamide and ammonia has been previously observed in similar systems involving zinc­(II) species. For example, the presence of acetamide was confirmed by X-ray crystallographic analysis of a cocrystal with the composition pipeamH[Zn(quin)_2_(CH_3_COO)]·acetamide (pipeamH^+^ = protonated piperidinoacetamidine, quin^–^ = quinaldinate).^9^ The in situ generated ammonia acted either as a ligand, forming [Zn­(quin)_2_(NH_3_)][Bibr ref20] and [Zn­(NH_3_)_4_]­SO_4_·H_2_O [unpublished results], or as a nucleophile, reacting with unhydrolyzed acetonitrile to yield acetamidine, CH_3_C­(NH_2_)=NH, which also coordinated to zinc­(II).^17^ All of these systems share common features: solvothermal conditions and the presence of methanol. Acetonitrile hydrolysis has also been applied in coordination chemistry by other research groups. For instance, during a solvothermal synthesis at 130 °C, hydrolysis products, acetate and acetamide, coordinated to copper­(II), leading to the formation of a characteristic copper­(II) acetate paddle-wheel motif with acetamide as axial ligands.^18^

During the search for better-diffracting crystals of **1**, various crystallization techniques were employed, and reaction conditions were systematically modified. Unfortunately, none of these methods yielded the desired outcome. One reaction system, however, is noteworthy. A reaction between zinc­(II) oxide and furosemide in a mixture of acetonitrile and methanol, left undisturbed for several months, resulted in the formation of new furosemide derivative **2**. Owing to its crystallization in the mixture of other compounds, its identity was determined solely by X-ray structural analysis on a single crystal. The ORTEP drawing of **2** is shown in Figure 3.

**Figure 3:**
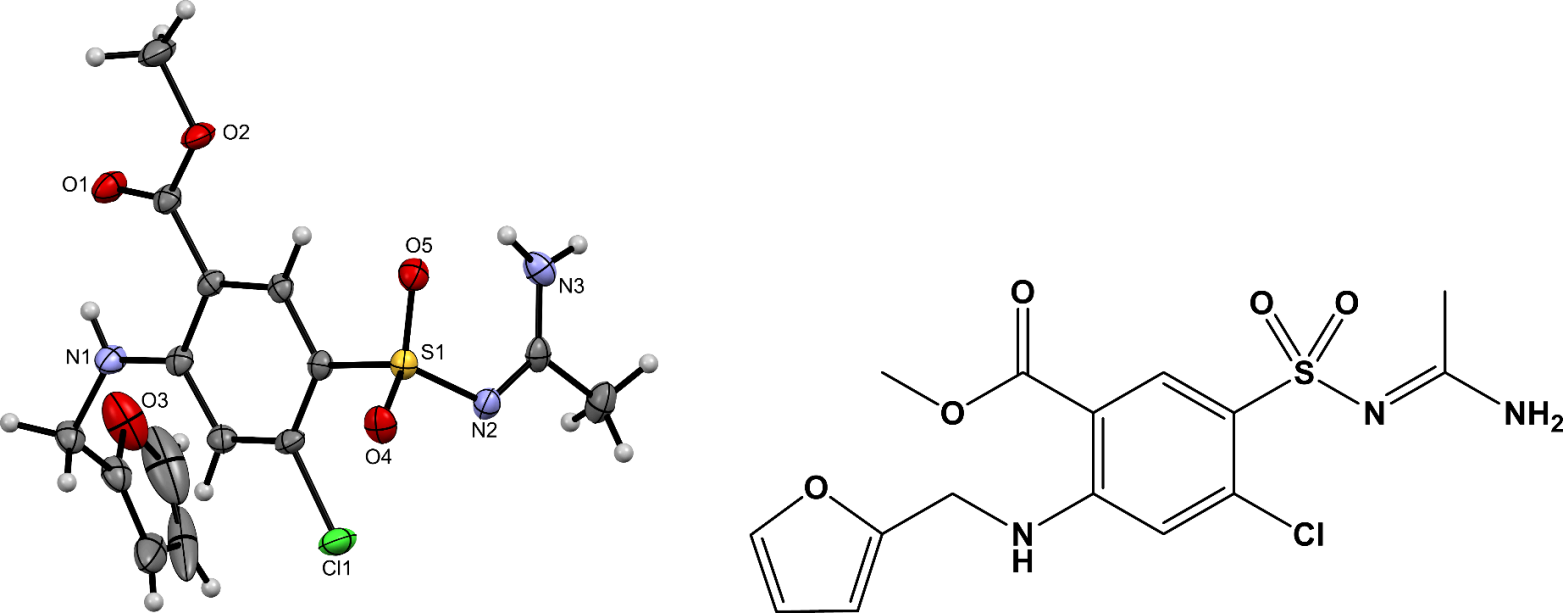
ORTEP drawing of a new furosemide derivative **2** with amidine and ester functional groups (left) and its structural formula (right). The displacement ellipsoids are shown at the 50% probability level. Hydrogen atoms are shown as spheres of arbitrary radii.

Compared to the parent furosemide, this derivative exhibits two notable structural modifications: the presence of both ester and amidine functional groups. These features result from two chemical transformations that occurred in the “ZnO-furosemide-acetonitrile-methanol” reaction system: (i) a nucleophilic attack of the sulfonamide NH_2_ group to the acetonitrile CN group, and (ii) esterification of the carboxyl group with methanol. Given that several furosemide esters are already known,^21^ the latter reaction is unsurprising. On the other hand, the reaction of sulfonamide with acetonitrile to form *N*-sulfonyl amidine is far less common. This is the first case in our laboratory where a primary amine reacts with a nitrile. Previously, only secondary amines such as piperidine and its derivatives were observed to undergo this transformation.^9,17^ Some amidines can exist in two tautomeric forms, A and B (Scheme 2). X-ray analysis showed that compound **2** adopts the type A tautomeric form in the solid state. Namely, the hydrogen atoms bonded to the nitrogen were located directly from the residual electron density map during structure refinement. The C–N bond lengths, which are nearly identical (1.316(3) and 1.322(2) Å), are consistent with the delocalization of the electron pair across the N–C–N fragment. The observed bond lengths are comparable to those reported in literature for *N*-sulfonyl amidines in tautomeric form A (C–N bond lengths in the range 1.316(4)–1.332(2) Å, the biggest difference between the C–N bonds was 0.012 Å).^22^ A series of recently prepared unsubstituted *N*-sulfonyl amidines was also found to crystallize exclusively in this tautomeric form.^22^ The existence of this form appears to be more general, as it has been observed in monosubstituted *N*-arylamidinates^23^ whose formation was attributed to a 1,3-hydrogen shift. The molecular structure of **2** is further stabilized by two relatively short intramolecular hydrogen bonds (ESI, Table S2). In the crystal lattice, intermolecular N–H···O hydrogen bonds between the amidine NH_2_ and the SO_2_ group link molecules into infinite supramolecular chains that propagate along the *a*-axis (ESI, Figure S4). Each chain has a symmetry-related counterpart *via* the center of inversion. These chains pack so that they are intertwined with each other (ESI, Figure S5). The observed hydrogen bonding motif further supports the tautomeric form A in the solid state, as both hydrogens from the NH_2_ group form hydrogen bonds. Although unsubstituted *N*-sulfonyl amidines are well documented and are of interest for their biological activity,^24,25^ the amidine derivative of furosemide has not been reported prior to this study. Many methodologies for the synthesis of *N*-sulfonyl amidines rely on azides.^26–30^ The synthesis of **2** is remarkably different. Namely, amidine results from a “direct” reaction of acetonitrile with the NH_2_ of sulfonamide, with 100% atom economy. Such a conversion is known to require catalytic amounts of zinc­(II) and elevated temperatures.^9,17^ The lower nucleophilicity of the sulfonamide group vs that of amine^31^ underscores the significance of the formation of **2**. Despite its appealing atom economy, the reaction system described here has a serious drawback, as compound **2** forms part of a complex mixture of unidentified solids. Further studies are required to find a rational synthetic pathway for novel furosemide derivative **2**.

**Scheme 2:**
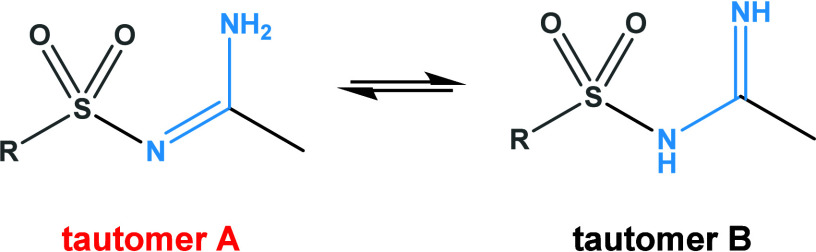
Two Tautomeric Forms of Amidines

In conclusion, the reaction systems involving zinc and furosemide featured several transformations: hydrolysis of acetonitrile with the formation of ammonia, which subsequently coordinated to zinc­(II) ions; esterification of the carboxylic acid with methanol; and nucleophilic addition of the amine from the sulfonamide group to the nitrile triple bond, leading to the formation of the corresponding *N*-sulfonyl amidine. This work describes the first amidine derivative of furosemide and reveals the unexpected reactivity of the sulfonamide group toward nitriles under solvothermal conditions.

## Methods

### General

Reagents were obtained from commercial sources and used as received. Acetonitrile was dried over molecular sieves prior to use.^32^ IR spectrum was recorded using a Bruker Alpha II FTIR spectrophotometer with an attenuated total reflection (ATR) module in the 4000–400 cm^–1^ range. The intensity of the bands is indicated as follows: w = weak, m = medium, s = strong, vs = very strong, and vvs = very very strong. ^1^H NMR spectrum was recorded on a Bruker Avance NEO 600 MHz instrument in deuterated dimethyl sulfoxide ((CD_3_)_2_SO) with 0.03% tetramethylsilane (TMS) standard. The residual solvent peak of (CD_3_)_2_SO at 2.50 ppm was used as a reference for the chemical shifts.^33^ The chemical shifts (δ) are given in ppm and the coupling constants (*J*) in Hz. Multiplicities are denoted as follows: s = singlet, d = doublet, t = triplet, q = quartet, and m = multiplet. The spectrum was processed using MestReNova software (version 14.2.2).^34^

### Thermal Analysis

The TG measurements were carried out with a Mettler Toledo TGA/DSC1 instrument in a temperature range from 25 to 250 °C. The heating rate was 10 K/min. During the measurement, the furnace was purged with nitrogen at a flow rate of 50 mL/min. 150 μL platinum crucibles were used, and the initial sample mass was 4.5095 mg in the case of the coupled TG-MS experiment, while for TG-FTIR, the setup was the same, and the initial sample mass was 5.3110 mg. For the TG curves, the blank curve was subtracted.

### TG-MS Analysis

Evolved gases were fed into a mass spectrometer (Pfeiffer Vacuum ThermoStar) via a 75 cm long heated transfer line. To reduce the water content in the mass spectrometer, the sample was kept at 30 °C for 20 min at the beginning of the measurement. Signals in the range of 2 to 90 *m*/*z* were collected, of which only the selected ones are shown.

### TG-FTIR Analysis

The coupling capillary was heated to 180 °C and connected to a Nicolet 6700 FTIR spectrometer (Thermo Scientific). The sample cell was maintained at 185 °C. The FTIR instrument was configured to continuously collect background-corrected spectra over a wavenumber range of 4000–400 cm^–1^ for the duration of the temperature program. Each spectrum represents an average of 5 scans with a resolution of 4 cm^–1^.

### X-ray Structure Analysis

Single-crystal XRD data were obtained using an Agilent SuperNova diffractometer with a molybdenum (Mo Kα, λ = 0.71073 Å) microfocus X-ray source at 150 K. CrysAlis PRO^35^ was used for data processing. Crystal structures were solved using the methods implemented in ShelXT^36^ within the Olex^2^ software.^37^ Refinement of the crystal structure was carried out using the least-squares methods in ShelXL.^38^ Anisotropic displacement parameters were determined for all nonhydrogen atoms. Due to the limited quality of the data for **1**, all hydrogen atoms were calculated. For **2**, hydrogen atoms on heteroatoms were located from the residual electron density and isotropically refined. The remaining hydrogen atoms were added in calculated positions. Data analysis was performed using Platon,^39^ and images were drawn with Mercury.^40^ Both crystal structures were deposited with the Cambridge Crystallographic Data Centre (CCDC) and assigned the deposition numbers: 2479073 (**1**) and 2479074 (**2**). The crystallographic data for **1** and **2** are summarized in Table S1.

### Preparation of [Zn­(fur)_2_(NH_3_)_2_]·(CH_3_CH_2_)_2_O (**1**)

A Teflon container was loaded with zinc­(II) oxide (50 mg, 0.61 mmol), furosemide (812 mg, 2.46 mmol), acetonitrile (5 mL), and methanol (5 mL). The container was closed and inserted into a steel autoclave, which was heated for 24 h at 105 °C. Afterward, the reaction mixture was allowed to cool slowly to room temperature and was then filtered. The filtrate was concentrated under reduced pressure on a rotary evaporator, and a glass vial with diethyl ether was carefully inserted into the Erlenmeyer flask with the concentrate. After about a week, single crystals of [Zn­(fur)_2_(NH_3_)_2_]·(CH_3_CH_2_)_2_O (**1**) were obtained. IR (ATR, cm^–1^): 3407w, 3365w, 3345w, 3284w, 2976w, 2928w, 2881w, 1609s, 1563s, 1497m, 1449w, 1382s, 1325vs, 1300s, 1270m, 1250m, 1226m, 1184w, 1157vvs, 1127m, 1105m, 1072m, 1058w, 1011m, 976w, 944s, 884w, 829m, 803m, 725s, 684s, 649m, 625m, 589vvs, 550s, 519s, 508s, 437m. ^1^H NMR ((CD_3_)_2_SO with 0.03% v/v TMS, 600 MHz): δ 9.45 (t, *J* = 6.0 Hz, 2H, N*H* fur^–^), 8.50 (s, 2H, C*H* fur^–^), 7.58 (s, 2H, C*H* fur^–^), 6.88 (s, 2H, C*H* fur^–^), 6.39–6.37 (m, 2H, C*H* fur^–^), 6.32 (d, *J* = 3.2 Hz, 2H, C*H* fur^–^), 4.47 (d, *J* = 6.0 Hz, 4H, C*H*_2_ fur^–^), 3.38 (q, *J* = 7.0 Hz, 2.8 H, (CH_3_C*H*_2_)_2_O), 1.09 (t, *J* = 7.0 Hz, 4H, (C*H*_3_CH_2_)_2_O) ppm. Elemental analysis calcd. for C_28_H_36_Cl_2_N_6_O_11_S_2_Zn (%): C, 40.37; H, 4.36; N, 10.09. Found (%): C, 39.52; H, 3.74; N, 10.04. *Note*. Deviations between experimental and theoretical values are attributed to a partial loss of solvent molecules upon removal of the product from the mother liquor.

### Preparation of **2**

A Teflon container was loaded with zinc­(II) oxide (50 mg, 0.61 mmol), furosemide (406 mg, 1.23 mmol), acetonitrile (5 mL), and methanol (5 mL). The container was closed and inserted into a steel autoclave, which was heated for 24 h at 105 °C. Afterward, the reaction mixture was allowed to cool slowly to room temperature and was then filtered. The resulting brown solution was stored at 4 °C for approximately two months. No precipitation occurred in the filtrate, so it was concentrated under reduced pressure on a rotary evaporator, and a glass vial with diethyl ether was carefully inserted into the Erlenmeyer flask with the concentrate. After a longer period at ambient conditions, single crystals of a new furosemide derivative **2** were obtained. *Note*. Compound **2** was analyzed only by X-ray structural analysis. Other characterization methods were not possible because compound **2** crystallized in the mixture.

## Supplementary Material



## Notes

X-ray structure analysis (Tables S1 and S2, Figures S1–S5), TG-FTIR analysis (Figure S6), Infrared spectrum of **1** (Figure S7), and ^1^H NMR spectrum of **1** (Figure S8) (PDF)
