# Supplementary material for: By Chance, Not Design: A New Furosemide Derivative From Zinc(II) Complex Studies
Source: ACS Omega. 2026 Feb 5;11(6):10614–9. doi: 10.1021/acsomega.5c12211 (PMC12917693; doi:10.1021/acsomega.5c12211)
Supplement: Supplementary file 1 [file ao5c12211_si_001.pdf]

Supporting Information
for

By chance, not design: A new furosemide derivative from zinc(II) complex studies

Nina Podjed Rihtaršič, Romana Cerc Korošec, Barbara Modec *

Faculty of Chemistry and Chemical Technology, University of Ljubljana, Večna pot 113, 1000 Ljubljana, Slovenia

* Corresponding author. E-mail: barbara.modec@fkkt.uni-lj.si

Contents

1. X-ray structure determinations
2. TG-FTIR analysis
3. Infrared spectroscopy
4. ^{1}H NMR spectroscopy
5. References

1. X-ray structure determinations

Table S1. Crystallographic data for **1** and **2**.

	**1** [a]	**2**
**Empirical formula**	$C_{28}H_{36}Cl_2N_6O_{11}S_2Zn$	$C_{15}H_{16}ClN_3O_5S$
**Formula weight**	833.02	385.82
**Crystal system**	monoclinic	triclinic
**Space group**	$P\,2_1/c$	$P\,-1$
$\boldsymbol{T}$ **[K]**	150.00(10)	150.00(10)
$\boldsymbol{\lambda}$ **[Å]**	0.71073	0.71073
$\boldsymbol{a}$ **[Å]**	18.5444(13)	7.1492(4)
$\boldsymbol{b}$ **[Å]**	10.7890(9)	10.9983(5)
$\boldsymbol{c}$ **[Å]**	18.4063(12)	12.0978(6)
$\boldsymbol{\alpha}$ **[°]**	90	100.194(4)
$\boldsymbol{\beta}$ **[°]**	108.360(7)	106.119(4)
$\boldsymbol{\gamma}$ **[°]**	90	107.458(5)
$\boldsymbol{V}$ **[Å^3]**	3495.2(5)	835.85(8)
$\boldsymbol{Z}$	4	2
$\boldsymbol{D}_{calc}$ **[g/cm^3]**	1.583	1.533
$\boldsymbol{\mu}$ **[mm^{-1}]**	1.041	0.386
**Collected reflections**	19476	12185
**Unique reflections**	6828	4555
**Observed reflections**	4642	3551
$\boldsymbol{R}_{int}$	0.0654	0.0441
$\boldsymbol{R}_1$ **($I > 2\sigma(I)$)**	0.1202	0.0476
$\boldsymbol{wR}_2$ **(all data)**	0.3609	0.1350

[a] Low quality data.

Figure S1. Overlay of the [Zn(fur)$_2$(NH$_3$)$_2$] complex molecules found in **1** (blue) and in an already known coordination compound (grey)[S1]. The overlay was done in Mercury.[S2] As shown in the overlay, the complex molecules differ in the relative orientation of the deprotonated furosemide ligands.

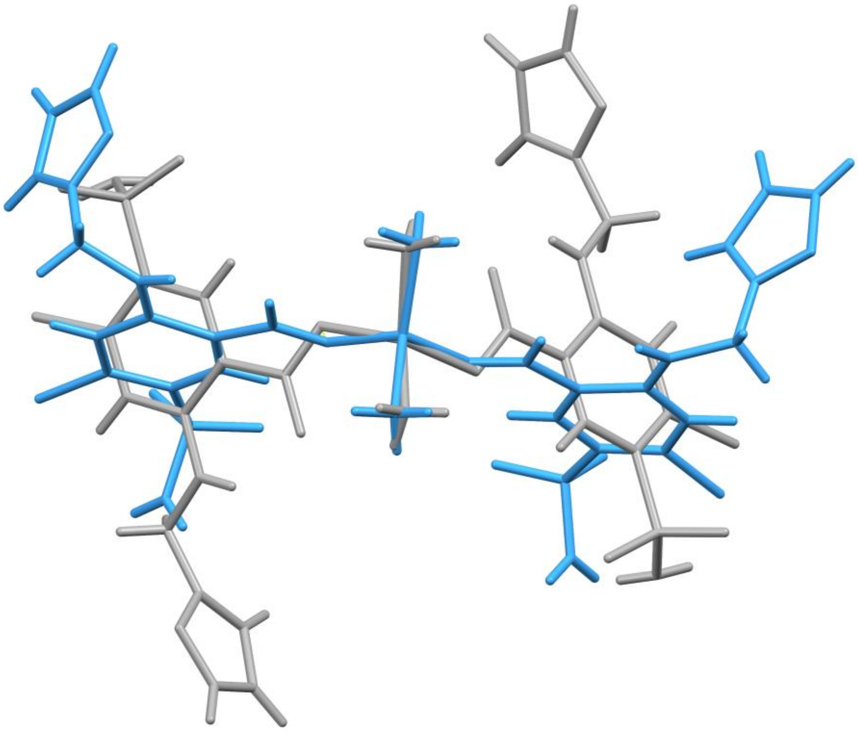

Figure S2. Hydrogen bonding pattern in $[Zn(fur)_2(NH_3)_2]\cdot(CH_3CH_2)_2O$ (**1**). Complex molecules are linked into supramolecular layers. A view perpendicular to the layer. Diethyl ether molecules are omitted.

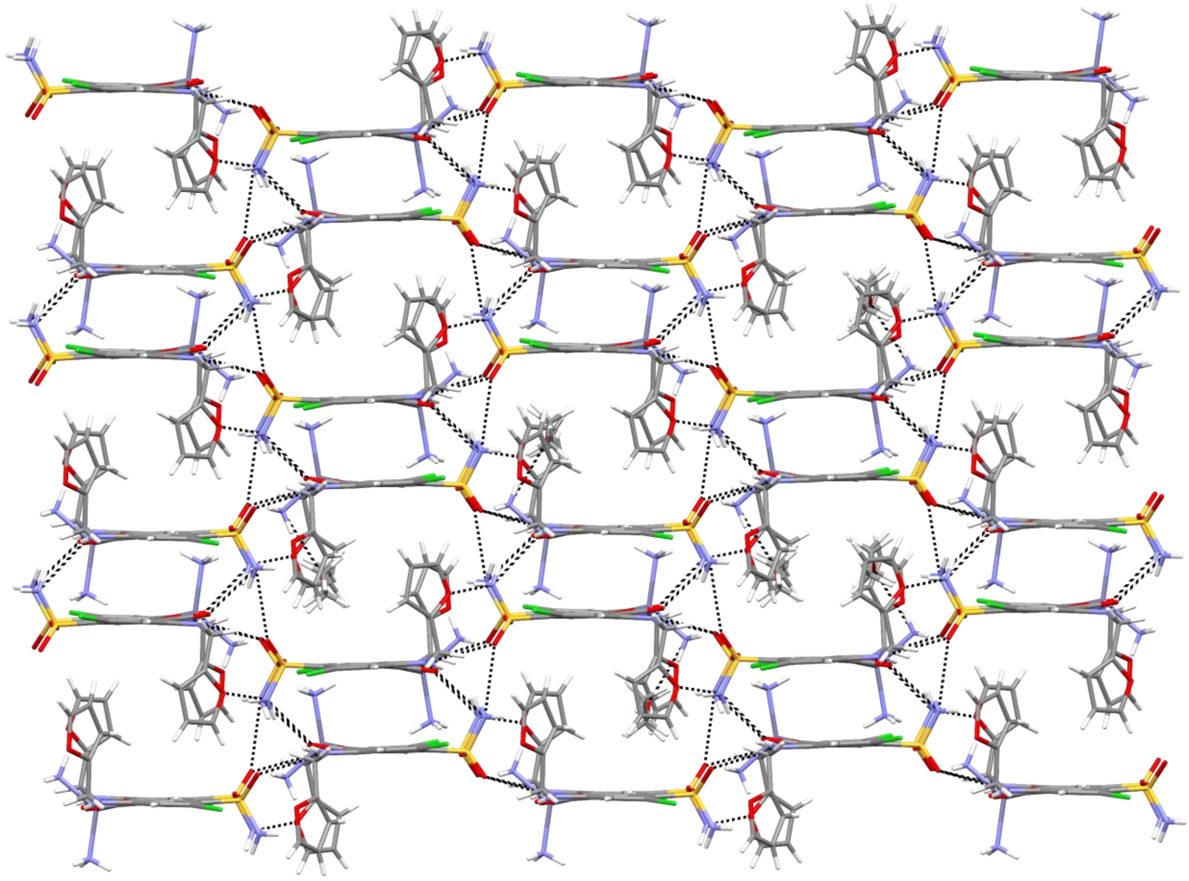

Figure S3. Supramolecular layers in **1** stack along *a*-axis. A view along the layers. Complex molecules are coloured grey and diethyl ether is red.

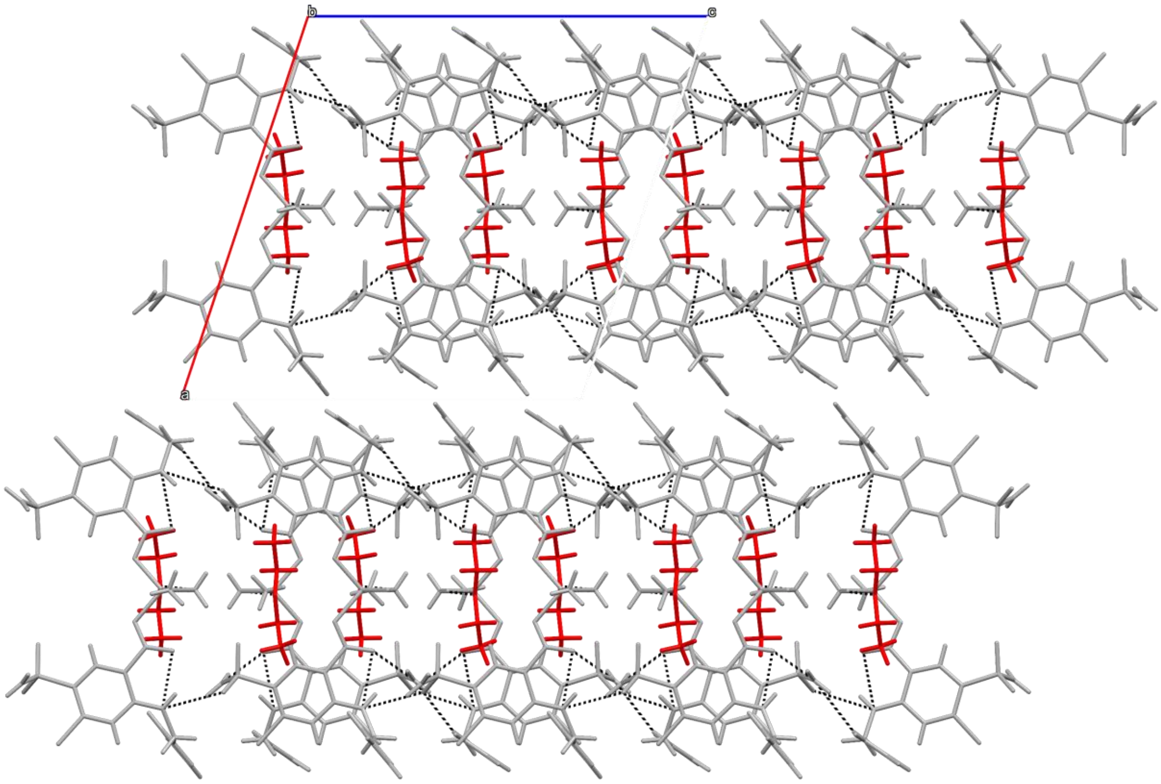

Table S2. Hydrogen bond parameters for **2**.

**Hydrogen bond**	**D···A distance [Å]**
NH···C=O(ester) [a]	N···O = 2.682(2)
NH_2(amidine)···SO_2 [a]	N···O = 2.763(2)
NH_2(amidine)···SO_2	N···O [–1+*x*, +*y*, *z*] = 2.939(2)

[a] Intramolecular hydrogen bond.

Figure S4. In **2**, hydrogen bonds link molecules into supramolecular chains. A short section of such a chain is shown.

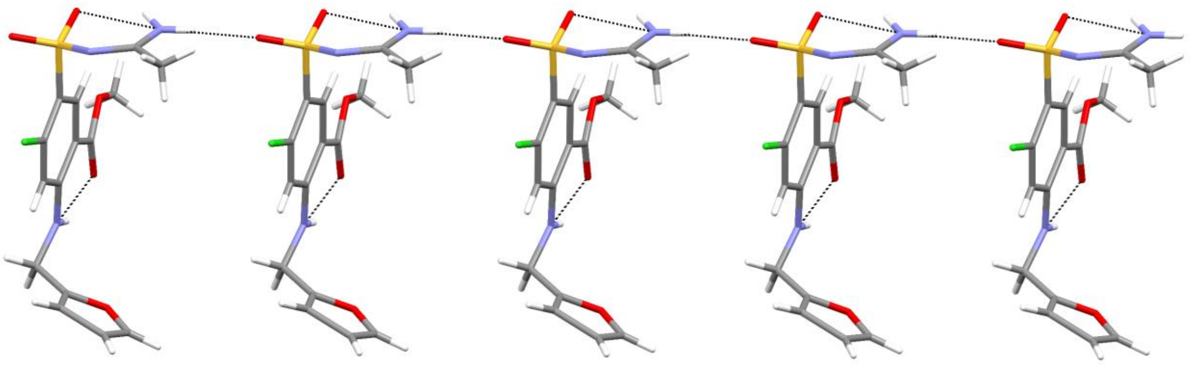

Figure S5. Packing of two chains in the structure of **2**. One chain is coloured grey, while its symmetry-counterpart is blue.

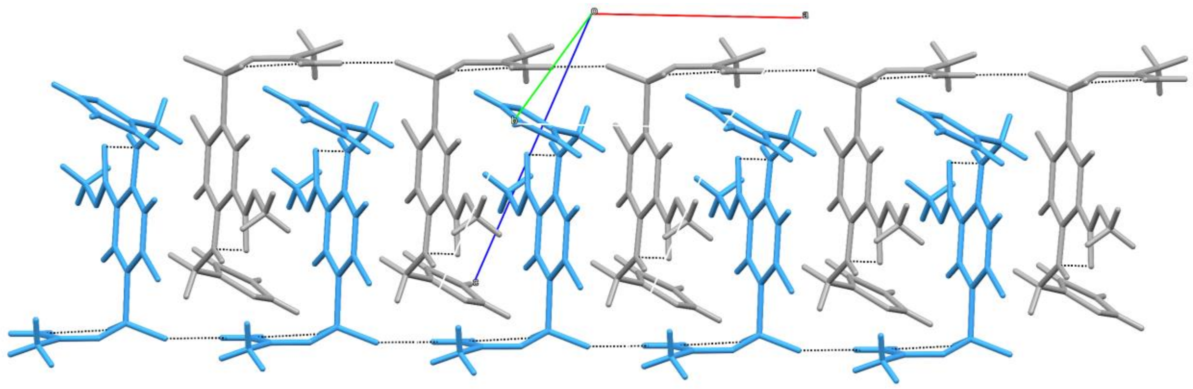

2. TG-FTIR analysis

Figure S6. a) TG and Gram-Schmidt curves, b) FTIR spectra measured during the second and third mass loss and the comparison with the ammonia gas spectrum from the NIST database.[S3]

The black curve in a) shows the TGA curve and the grey one the Gram-Schmidt curve, which represents the total infrared intensity of the collected spectrum at a specific point in time. Figure S6 b) shows the FTIR spectra, recorded at the maxima of the Gram-Schmidt curve during the second and third mass loss (after 11.75 min and 15.58 min). The comparison with the database shows that these bands are typical for ammonia.

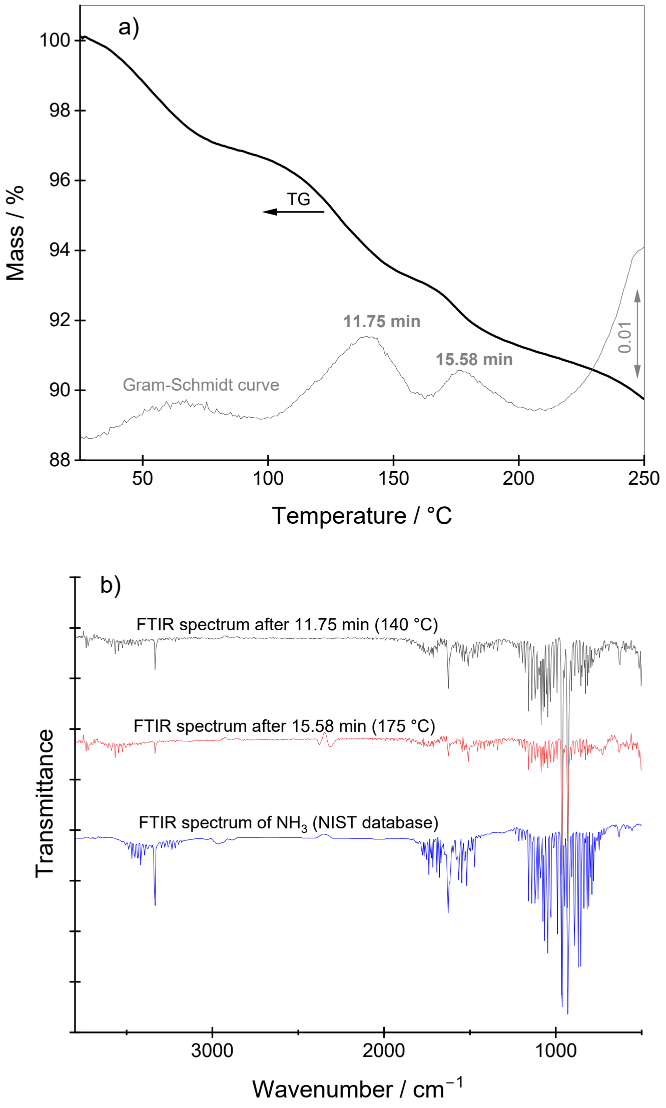

3. Infrared spectroscopy

Figure S7. Infrared spectrum of $[Zn(fur)_2(NH_3)_2]\cdot(CH_3CH_2)_2O$ (**1**).

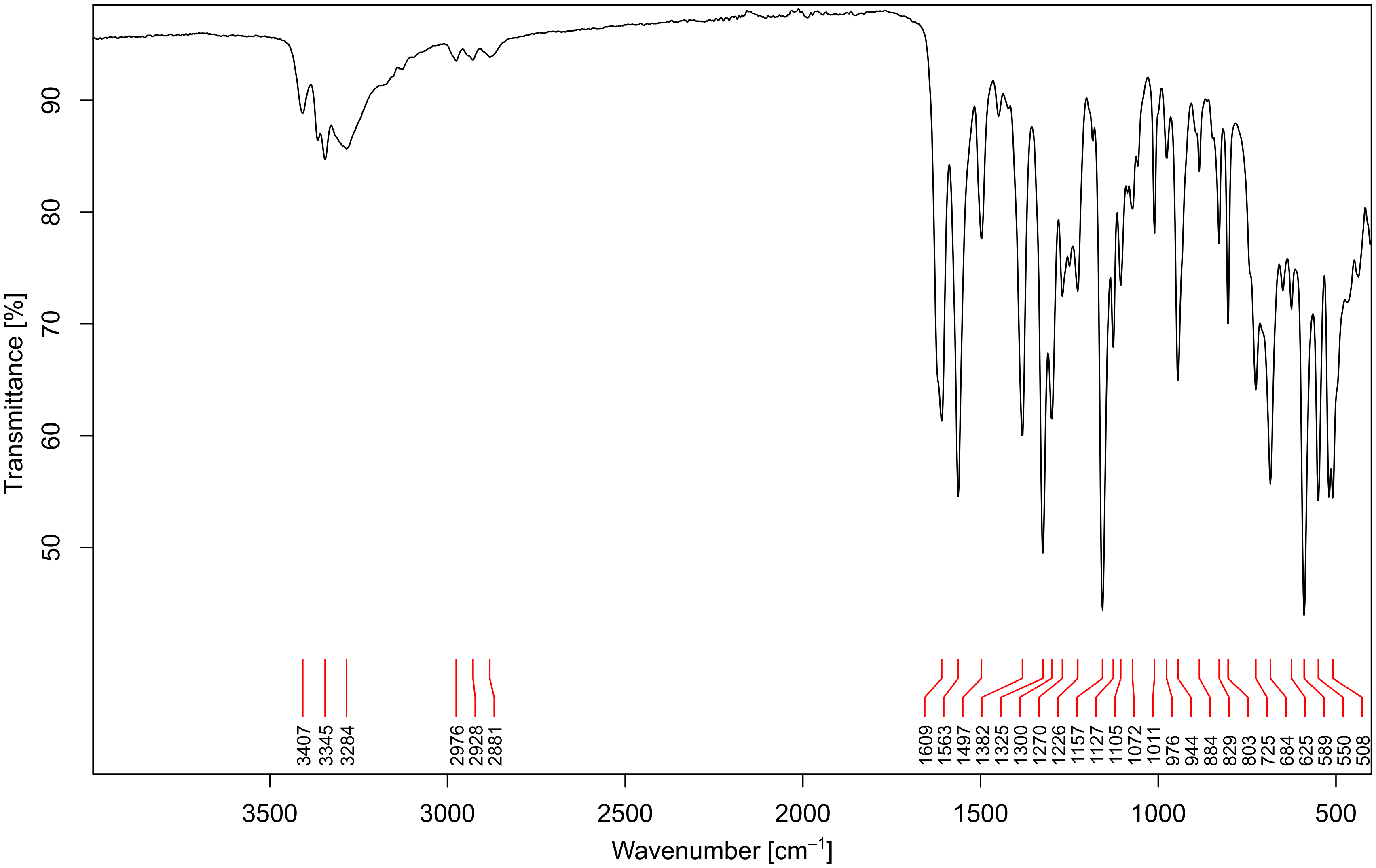

4. ^{1}H NMR spectroscopy

Figure S8. ^{1}H NMR spectrum of [Zn(fur)$_2$(NH$_3$)$_2$]·(CH$_3$CH$_2$)$_2$O (**1**) in DMSO-d_6.

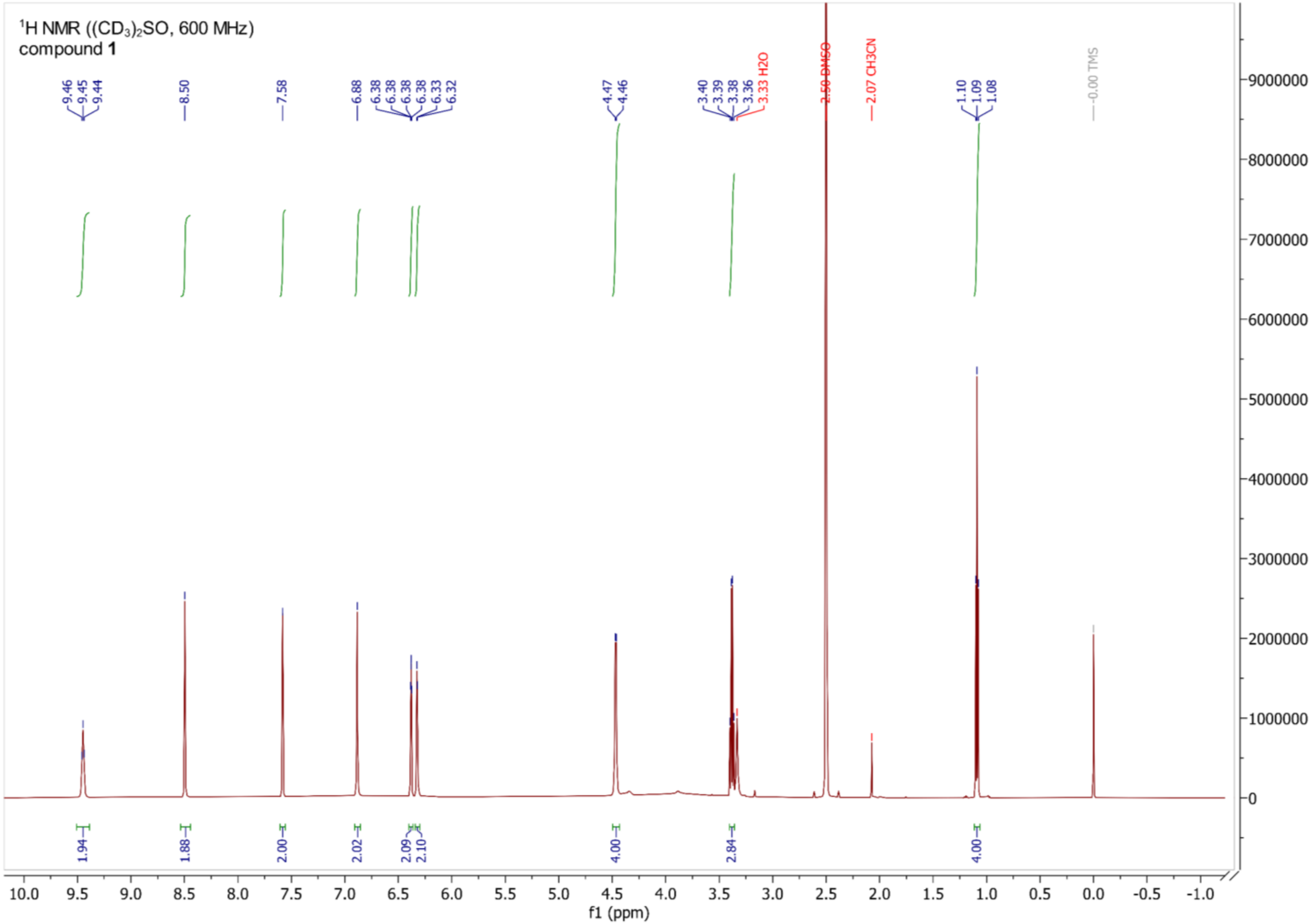

5. References

(S1) Podjed, N.; Uranjek, Z.; Cerc Korošec, R.; Hrast Rambaher, M.; Golob, M.; Modec, B. On zinc(II) coordination chemistry with furosemide: a journey from a mononuclear complex to a coordination polymer. *New J. Chem.* **2025**, *49*, 9113–9122.
(S2) Macrae, C. F.; Sovago, I.; Cottrell, S. J.; Galek, P. T. A.; McCabe, P.; Pidcock, E.; Platings, M.; Shields, G. P.; Stevens, J. S.; Towler, M.; et al. *Mercury 4.0*: from visualization to analysis, design and prediction. *J. Appl. Cryst.* **2020**, *53*, 226–235.
(S3) NIST Mass Spectrometry Data Center, W. E. Wallace, director. Mass Spectra. In *NIST Chemistry WebBook, NIST Standard Reference Database Number 69*, Linstrom, P. J., Mallard, W. G. Eds.; National Institute of Standards and Technology, Gaithersburg MD, 20899.
